# The associations between Parkinson’s disease and cancer: the plot thickens

**DOI:** 10.1186/s40035-015-0043-z

**Published:** 2015-10-26

**Authors:** Danielle D. Feng, Waijiao Cai, Xiqun Chen

**Affiliations:** MassGeneral Institute for Neurodegenerative Disease, Department of Neurology, Massachusetts General Hospital, Harvard Medical School, Boston, MA USA; Department of Epidemiology, Harvard T.H. Chan School of Public Health, Boston, MA USA; Key Laboratory of Cellular and Molecular Biology, Huashan Hospital, Fudan University, Shanghai, 200040 China

## Abstract

Epidemiological studies support a general inverse association between the risk of cancer development and Parkinson’s disease (PD). In recent years however, increasing amount of eclectic evidence points to a positive association between PD and cancers through different temporal analyses and ethnic groups. This positive association has been supported by several common genetic mutations in *SNCA*, *PARK2*, *PARK8*, *ATM*, *p53*, *PTEN*, and *MC1R* resulting in cellular changes such as mitochondrial dysfunction, aberrant protein aggregation, and cell cycle dysregulation. Here, we review the epidemiological and biological advances of the past decade in the association between PD and cancers to offer insight on the recent and sometimes contradictory findings.

## Introduction

Parkinson’s disease (PD) is one of the most common and rigorously studied age-related neurodegenerative disorders, occurring in 0.3 % of the whole population and nearly 2 % in those over 65 years of age in industrialized countries [1]. Resting tremor, rigidity, hypokinesia, and postural instability are the four cardinal motor symptoms of PD resulting from the loss of dopaminergic neurons in the substantia nigra pars compacta. While the prevalence of this slowly debilitating disease is increasing, it remains incurable and irreversible due to its elusive mechanisms. Another chronic disease devastating human health and of substantial research focus is cancer. Many epidemiological studies have reported associations between PD and cancers, supporting a general inverse and more recently, positive association in certain cancers including skin, breast, and brain. This positive association is corroborated by advances in molecular genetics and cell biology revealing several genetic mutations that alter cell cycle control, protein turnover, and mitochondrial functions. This intriguing association between PD and cancers provides a new perspective to the well-known opposing cell fates of degeneration and death of post-mitotic neurons, and the uncontrolled division and enhanced resistance to death of cancer cells. The convergence of these processes provides new avenues to study both of the age-related conditions and address an urgent need for therapeutic options.

## Epidemiological associations between PD and cancer

### General trends between PD and common cancers

Many epidemiological studies have indicated an inverse association between the risk of developing cancers and PD. In a meta-analysis of 29 studies by Bajaj et al. that included 107598 PD patients, the diagnosis of PD was associated with an overall 27 % decreased risk of all cancers included in the studies, and 31 % decreased risk after exclusion of melanoma and other skin tumors [2]. Similarly, a recent 2014 meta-analysis by Catala-Lopez et al. found 17 % decreased risk of cancer in PD patients [3]. The most widely reported reduced risks in PD patients are cancers of the prostate, lung, bladder, stomach, colorectal, blood, and uterus (Table 1). While the lower risks of lung, bladder, and colorectal cancer, all smoking-related cancers, in PD patients are generally undisputed, stomach, leukemia, and uterine cancers fail to achieve significance in some studies for a clear inverse association.

**Table 1:** Representative epidemiological studies of PD-cancer from 1995-2015

Study	Reported positive association			Reported negative association						
	Breast	Non-melanoma skin	Brain	Prostate	Lung	Bladder	Stomach	Colorectal	Leukemia	Uterus
Lin et al., 2015 [7]	1.11 (0.90–1.37)^c^	**1.81 (1.46–2.23)** ^c^	**3.42 (1.84–6.38)** ^c^	***1.80 (1.52–2.13)*** ^*c*^	***1.56 (1.38–1.76)*** ^*c*^	***1.59 (1.25–2.01)*** ^*c*^	***1.59 (1.30–1.94)*** ^*c*^	***1.47 (1.31–1.65)*** ^*c*^	***1.62 (1.31–2.01)*** ^*c*^ ^e^	***1.83 (1.12–3.01)*** ^*c*^
Ong et al., 2014 [6]	**1.16 (1.1 0–1.22)** ^c^	***0.89 (0.86–0.92)*** ^*c*^	**1.50 (1.34–1.68)** ^c^	0.98 (0.94–1.01) ^c^	**0.75 (0.71–0.78)** ^c^	**0.86 (0.82–0.91)** ^c^	**0.87 (0.80–0.95)** ^c^	**Colon: 0.87 (0.83–0.91)** ^c^	*Lymphatic: 1.11 (1.00–1.23)* ^*c*^	***Corpus: 1.17 (1.03–1.32)*** ^*c*^
								**Rectum: 0.89 (0.83–0.97)** ^c^	**Myeloid: 0.82 (0.72–0.94)** ^c^	*Cervix*: *1.09 (0.83–1.40)* ^c^
Wirdefeldt et al., 2014 [5]	1.02 (0.86, 1.21)^a^	1.05 (0.82, 1.36)^a^	1.43 (0.95, 2.14)^a^	*1.12 (0.96– 1.31)* ^a^	*1.11 (0.60–2.04)* ^*a*^	*1.15 (0.89-1.48)* ^*a*^	0.61 (0.24–1.54)^a^	Colon: 0.75 (0.54–1.04)^a^	*Lymphatic:* 0.78 (0.35–1.76)^a^	***Corpus: 1.66 (1.21–2.28)*** ^a^
	1.24 (0.81–1.92) ^b^	1.54 (0.96–2.49)^b^	**4.78 (2.24–10.2)** ^b^	0.99 (0.76–1.29)^b^	*1.59 (0.92–2.77)* ^*b*^	*1.54 (0.95-2.50)* ^*b*^	*1.43 (0.66–3.08)* ^*b*^	**Colon: 1.66 (1.09–2.53)** ^b^	*Lymphatic: 1.78 (0.83–3.8)* ^*c*^	***Corpus: 2.55 (1.17–5.58)*** ^b^
	*0.80 (0.57, 1.12)* ^*c*^	**1.40 (1.04, 1.88)** ^c^	1.52 (0.86, 2.69)^c^	**0.77 (0.63–0.92)** ^c^	**0.4 (0.24–0.66)** ^c^	**0.40 (0.24-0.66)** ^c^		Colon: 0.74 (0.52, 1.05)^c^	***Myeloid: 2.95 (1.02–8.59)*** ^*b*^	Corpus: 0.51 (0.23–1.13)^c^
Rugbjerg et al., 2012 [15]	**1.17 (1.02–1.34)** ^c^	**1.29 (1.18–1.39)** ^c^	*0.99 (0.67–1.40)* ^*c*^	**0.74 (0.64–0.86)** ^c^	**0.40 (0.33–0.48)** ^c^	**0.48 (0.38–0.60)** ^c^		**0.82 (0.73–0.92)** ^c^	**Lymphatic: 0.66 (0.42–0.99)** ^c^	Corpus: 0.82 (0.58–1.13)^c^
Kareus et al., 2012 [11]				***1.71 (1.49–1.96)***	**0.22 (0.09–0.43)**		**0.22 (0.03–0.78)**	**0.55 (0.37–0.79)**		
Fois et al., 2010 [13]	*0.9 (0.7 to 1.0)* ^a^	1.0 (0.8 to 1.1)^a^	1.0 (0.4 to 2.1)^a^	0.9 (0.7 to 1.1)^a^	**0.5 (0.4 to 0.7)** ^a^	**0.7 (0.6 to 0.9)** ^a^	0.8 (0.5 to 1.1)^a^	**Colon: 0.7 (0.6 to 0.9)** ^a^	0.7 (0.4 to 1.2)^a^	0.9 (0.6 to 1.3)^a^
	*0.7 (0.4 to 1.0)* ^*c*^	***0.6 (0.3 to 0.9)*** ^*c*^	*0.8 (0.1–2.8)* ^*c*^	**0.7 (0.5 to 1.0)** ^c^	**0.5 (0.4 to 0.8)** ^c^	**0.5 (0.3 to 0.9)** ^c^	**0.6 (0.3 to 0.9)** ^c^	**0.5 (0.4 to 0.8)** ^c^	0.9 (0.4 to 1.6)^c^	0.8 (0.2 to 2.0)^c^
Lo et al., 2010 [166]	*0.72 (0.27–1.9)* ^a^ ^d^ *0.95 (0.38–2.4)* ^*c*^			*1.01 (0.47–2.2)* ^a^ ^d^	0.45 (0.05–4.5)^a^ ^d^	*1.03 (0.26–4.2)* ^*a*^ ^*d*^		0.61 (0.11–3.4)^a^ ^d^		
				0.80 (0.41–1.6) ^c^	0.35 (0.10–1.2)^c^	0.73 (0.24–2.2)^c^		0.66 (0.27–1.6)^c^		
Becker et al., 2010 [167]	*0.98 (0.53–1.80)* ^*c*^			0.86 (0.56–1.32)^c^	**0.47 (0.25–0.86)** ^c^			0.88 (0.48–1.63)^c^	**0.33 (0.18–0.61)** ^c^ ^e^	
Driver et al., 2007 [12]			*0.83 (0.14-4.96)* ^c^	0.74 (0.44–1.2)^c^	0.32 (0.07–1.53)^c^	0.68 (0.16–2.84)^c^		0.54 (0.14–2.16)^c^	0.81 (0.22-2.90)^c^	
Olsen et al., 2006 [4]	1.09 (0.90–1.33)^a^ ^d^	**1.26 (1.11–1.43)** ^a^ ^d^	*0.97 (0.55–1.70)* ^a^ ^d^	0.99 (0.75–1.31)^a^ ^d^	**0.42 (0.22–0.80)** ^a^ ^d^	**0.71 (0.55–0.91)** ^a^ ^d^	*1.03 (0.50–2.14)* ^a^ ^d^	***Colon: 1.29 (1.02–1.63)*** ^a^ ^d^	0.43 (0.19–1.01)^a^ ^d^	Cervix: 0.93 (0.66–1.31)^a^ ^d^
								Rectum: 0.98 (0.70–1.36)^a^ ^d^		
Olsen et al., 2005 [9]	**1.24 (1.0–1.5)** ^c^	**1.25 (1.1–1.4)** ^c^	1.32 (0.9–1.9)^c^	**0.74 (0.6–0.9)** ^c^	**0.38 (0.3–0.5)** ^c^	**0.52 (0.4–0.7)** ^c^	0.83 (0.6–1.1) ^c^	Colon: 0.84 (0.7–1.0)^c^	Myeloid: 0.69 (0.4–1.2)^c^	Cervix: 0.76 (0.4–1.4)^c^
								Rectum: 0.89 (0.7–1.1)^c^		
Elbaz et al., 2005 [168]		**1.76 (1.07–2.89)** ^c^								
Minami et al., 2000 [169]	**5.49 (1.10–16.03)**									
Moller et al., 1995 [10]	1.20 (0.9– 1.5)^c^	1.24 (1.0–1.5)^c^	1.61 (0.9–2.7)^c^	0.79 (0.6–1.1)^c^	**0.29 (0.2–0.4)** ^c^	**0.42 (0.2–0.7)** ^c^	0.91 (0.6– 1.4)^c^	Colon: 0.96 (0.7– 1.2)^c^	0.82 (0.4–1.4)^c^	Cervix: 0.86 (0.3– 1.9)^c^
								Rectum: 0.98 (0.7–1.4)^c^		Corpus: 0.89 (0.4– 1.6)^c^

Statistically significant values of relative risks (hazard and incidence rate ratios) according to authors’ thresholds are bolded. Associations that do not follow the general trend are highlighted in italics.

^a^Before PD diagnosis

^b^Within one year of PD diagnosis

^c^After PD diagnosis

^d^Odds ratios

^e^Lymphoma or leukemia

Evidence also links PD to an increased prevalence of a few cancers (Table 1), in particular non-melanoma skin cancer. Associations of breast and brain cancers with PD are suspected but conflicting, as is the temporal association between cancers and PD occurrence. For instance, Olsen et al. found that individuals before PD diagnosis have significant risk of non-melanoma skin cancer [4], but is countered by a recent study by Wirdefeldt et al. showing increased risk of 40 % only after 1 year following PD diagnosis [5]. Likewise, other studies that investigate the risk of cancer before and after PD diagnosis reveal associations that differ in direction and statistical significance.

Trends differ across ethnicities and between Eastern and Western populations. In two Taiwanese and British cohort studies, brain, kidney, and uterine cancers were positively associated with PD after diagnosis, but stomach and lung cancer were negatively associated with PD in the British study [6], while they were positive in the Taiwanese study [7]. In fact, the authors of the Taiwanese study did not report negative risks for any cancer in PD patients. The validity of these differences, whether due to differential genetic, environmental factors, or technic variations, should be closely examined, as a previously published Taiwanese matched cohort study of the same database found that lung cancer had lower hazard ratio in PD versus non-PD patients [8], corroborating the negative association of studies in Danish [9, 10], American [11, 12], Swedish [5], and British [13] populations. Further investigations of cancer in Asian PD patients are warranted.

### PD and melanoma

Melanoma has an established and consistently positive association with PD (Table 2). However, individual studies show relative risk with considerable variation from 0.5 to 20.9. Although the studies controlled for similar factors, including age, race, and sex, differences invariably arise from biases due to selection and inclusion criteria, diagnostic practices and confirmation of pathology, sample sizes, and unadjusted covariates. Smoking history and family history of melanoma or PD should be accounted for as confounders in the statistical models if possible. Smoking has long been associated with PD, and melanoma could introduce survival bias due to unrelated diseases and comorbidity. Similarly, there is an apparent genetic basis for the PD-melanoma association: individuals with first-degree family history of melanoma have 85 % higher relative risk of PD [14], and the association extends to third-degree relatives [11]. Melanoma is fairly uncommon amongst PD patients, as evidenced by 9 observed cases in a cohort of 806 PD patients of the PRECEPT study and 19 melanoma versus 1718 non-melanoma subjects of the NET-PD study, thereby allowing more room for variability in calculated risks and requiring greater statistical sensitivity in analyses. Furthermore, a temporal association seems to exist: melanoma risk increases closer to the index date of PD [4, 9, 15]. To consolidate some of these factors across studies, a meta-analysis of 12 studies report that the overall odds ratio for melanoma in people with PD versus no PD was 2.11 [16]. The authors also highlight that the odds ratio after PD diagnosis is 3.61 versus statistically insignificant 1.07 before PD diagnosis [16].

**Table 2:** Studies of melanoma risk in PD patients and vice versa

Melanoma risk in PD patients		
Relative risk	Measure of association, 95 % confidence interval	Study/Subjects
	**3.6 (2.2–5.6)** ^b^ [170]	National Institutes of Health Exploratory Trials in PD Long-term Study 1 (NET-PD)
	**2.24 (1.21–4.17)** ^b^ [171]	North American PD patients vs. US Surveillance Epidemiology and End Results cancer database, American Academy of Dermatology skin cancer screening programs
	**20.9 (9.6–39.7)** ^b^ [172]	Parkinson Research Examination of CEP-1347 Trial (PRECEPT)
	**1.95 (1.4–2.6)** ^b^ [9]	Danish National Hospital Register, Danish Cancer Registry
	1.70 (0.62–4.67)^b^ [167]	UK General Practice Research Database
	***0.5 (0.2–0.9)*** ^*a*^ [13]	Oxford Record Linkage Study
	**1.96 (1.1–3.2)** ^b^ [10]	Danish National Hospital Register, Danish Cancer Registry, Danish Register of Deaths
	**1.95 (1.44–2.59)** [11]	Utah Population Database, US Surveillance, Epidemiology and End Results
	**1.41 (1.09–1.80)** ^b^ [15]	Danish National Hospital Register, Danish Cancer Registry
	**6.15 (1.77–21.37)** ^b^ [12]	US Physicians Health Study
Odds ratio	**1.44 (1.03–2.01)** ^a^ [4]	Danish National Hospital Register, Danish Cancer Registry
	1.5 (0.40–5.2)^a^1.6 (0.71–3.6)^b^ [166]	Parkinsonism Epidemiology at Kaiser (PEAK)
PD risk in melanoma patients		
Mortality ratio	**266.3 (222–317)** [173]	Australian National Cancer Statistics Clearing House
Relative risk	**1.65 (1.22–2.19)** [11]	Utah Population Database, US Surveillance, Epidemiology and End Results

Statistically significant values of relative risks according to authors’ thresholds are bolded. Associations that do not follow the general trend are in italics.

^a^ Before PD diagnosis

^b^ After PD diagnosis

L-3,4-dihydroxyphenylalanine (L-DOPA), the main drug used to treat PD, was initially proposed to be responsible for the association with melanoma [17] although it has now been widely discredited [18, 19]. Several pigmentation interrelated proteins, including tyrosinase, tyrosine hydroxylase, melanin, and sphingolipids [20–22], and possible common risk factors including pesticide exposure [23] and lack of smoking, caffeine, and alcohol intake [24–29] have emerged to help explain the association. Tyrosine hydroxylase converts tyrosine to the dopamine precursor L-DOPA in both melanocytes and neurons while tyrosinase converts tyrosine to L-DOPA and dopaquinone, the precursor to pheomelanins and eumelanins [30]. Sorting of tyrosinase to melanosomes from the Golgi seems to require glycosphingolipids [31]. Dysregulation of glycosphingolipid metabolism, storage, and interaction with α-synuclein, as well as mutations of the phospholipase A2, group VI (*PLA2G6*) gene regulating synthesis of glycosphingolipids’ core component ceramide, are associated with both melanoma and PD [32–36]. Adding to the complexity, melanin, the end product of this pathway, could be a doubled-edged sword. Melanin serves to dissipate UV radiation and protect against DNA damage in skin cells, and neuromelanin binds to 1-methyl-4-phenylpyridinium ion (MPP^+^) and metal ions to reduce their toxicity [37]. Hence, a hypothesis put forward for the negative link between smoking and melanoma and PD is the stimulated production of melanin and neuromelanin by nicotine [38], although whether or not nicotine is truly beneficial is debatable as neuromelanin also activates microglia and stimulates their secretion of proinflammatory cytokines tumor-necrosis factor α, interleukin 6, and nitric oxide in a degenerate cycle [37].

## Genetic determinants and associated cellular processes shared in PD and cancer

A growing body of evidence supports common genetic mechanisms in cancer and neurodegenerative diseases. Mutations in a variety of genes involved in the dysregulation of the cell cycle and protein turnover have been implicated in both PD and cancer.

### SNCA

*SNCA* is perhaps the best known causal gene for PD, with several missense mutations (A53T, E46K, H50Q, G51D, A30T), gene duplications, or posttranslational modifications of α-synuclein that ultimately lead to its misfolding and aggregation of insoluble fibrils [39]. The function of α-synuclein has been postulated to facilitate the release of neurotransmitters at synapses, and recent evidence has shown support for this hypothesis. In mutated α-synuclein E57K mouse lines that accumulate oligomers, there were loss of synaptic terminals and dendrites and impairment of vesicle transport [40]. They exist in the unfolded monomeric form in the cytosol, but act as chaperones in the multimeric state when bound to plasma membrane or docked synaptic vesicles in the process of forming SNARE complexes, which are crucial for neurotransmitter release from the presynaptic membrane [41–43]. The soluble monomeric form, rather than the membrane bound α helical multimeric form, is the basis of aggregates and associated neurotoxicity [44]. The interaction with membranes is possibly stabilized through the positively charged N-terminus of α-synuclein binding to the negatively charged lipid membrane of vesicles through a lipid ordering effect [45, 46]. Interestingly, α-synuclein is now also implicated in reducing Aβ deposition and plaque formation in Alzheimer’s disease, although the interaction between α-synuclein and Aβ does not seem to attenuate, but rather exacerbate, synapse and dendritic loss [47].

α-synuclein is also linked to various cancers although the biological consequences are relatively unknown. Immunohistological studies revealed its expression in ependymoma, astrocytoma, breast and ovarian cancerous tissues, and these cancers co-occur with PD in epidemiological studies discussed previously [48, 49]. The methylated state of *SNCA* and the presence of α-synuclein in melanocytic lesions may be used as biomarkers for some lymphomas and melanoma [50, 51]. Although α-synuclein is a hallmark of PD, its propagation mechanism may help explain its appearance in cancers outside the brain and in human plasma and cerebrospinal fluid [52–54]. In melanoma, S129-phosphorylated form of α-synuclein, the pathological form in Lewy bodies of PD, but not the unphosphorylated form, is localized to the surface of melanoma cells and their released microvesicles’ membranes [55]. The exosomal secretion pathway, implicated in prion diseases, could also play a role. Neurons and astrocytes can secrete exosomes containing α-synuclein oligomers inside and on the surface of their membranes, conferring the oligomers greater chance of reuptake by another cell than when exosome-free [56, 57]. Strikingly, α-synuclein has recently been shown to cross the blood–brain barrier, which has increased permeability in PD, after intravenous injection in mice [58, 59]. Taken together, an emerging hypothesis for the aggregation and prion-like propagation of toxic α-synuclein present in PD and its co-appearance in brain and other cancers could be through its release and uptake via various mechanisms between neurons and cells outside of the central nervous system (CNS).

### PARK2

*PARK2* encodes parkin, which is a component of a multiprotein E3 ubiquitin ligase complex, a part of the ubiquitin-proteasome system (UPS) that mediates the targeting of proteins for degradation [60, 61]. Genetically, *PARK2* is the most commonly mutated gene in autosomal recessive PD, accounting for nearly 50 % of cases [62, 63]. In one study of early onset PD, mutations were detected in 77 % of patients aged 20 or younger [64]. Heterozygous mutations of *PARK2* in exon 7, the first RING finger (C253W, R256C, R275W, and D280N) act as susceptibility alleles for late onset PD [63, 65, 66]. In cancer, although the association between the exact alterations of *PARK2* and cancer susceptibility is not well understood, the mutations, deletion, copy number alterations, promoter hypermethylation, and aberrant mRNA and protein expression of *PARK2* have all been found to be prevalent across human malignancies, especially glioma, lung, breast, colon, and ovarian cancer [7, 67–70].

The precise mechanism by which mutations in *PARK2* lead to PD and cancer remains unclear. However, accumulation of cyclin E, a substrate of *PARK2*, seems to be a central event [71]. Cyclin E is the primary cyclin that drives S-phase progression [72]. Loss of function of *PARK2* mutations results in the deficiency of E3 ligase, leading to dysfunctional UPS, buildup of cyclin E and β-catenin, and upregulation of Wnt and EGFR-AKT pathways [60, 61, 73]. CDK2/cyclin E phosphorylates the tumor suppressor retinoblastoma, releasing the transcription factor E2F-1 from inhibition. In cycling cells, E2F-1 upregulates proteins facilitating progression through S phase, but in post-mitotic neurons, E2F-1 triggers apoptosis through p53 and Bax [74, 75]. Conversely, the overexpression of β-catenin, a protein overexpressed in many cancers, may partially explain why some cancers are inversely associated with PD, as signaling of Wnt/β-catenin promotes repairing mechanisms in dopaminergic neurons [76]. On the other hand, EGFR-AKT signaling is likely tissue specific and context dependent, as loss of *PARK2* have been found to increase or decrease the activity of EGFR-AKT, and subsequently, promote or hinder neuronal survival, adding a complex layer to the conflicting association between PD and different cancers [73, 77].

### PARK8

The *PARK8* gene encodes the leucine-rich repeat kinase 2 (LRRK2) protein, with the G2019S, R1441G/C/H, Y1699C, G2385R, R1628P, I2020T mutations appears to increase the risk of PD [78–80]. LRRK2 has been reported to have complex and extensive roles related its well-known functions as a GTPase and kinase, including regulation of synaptogenesis, axon and dendritic growth, and synaptic vesicle release and interaction with tau, 14-3-3 proteins, and the presynaptic proteins syntaxin 1A, synapsin I, dynamin I, and VAMP2 [81–86]. One of the most notable causal autosomal dominant mutations for PD, G2019S, was first discovered in Ashkenazi Jewish and North African Arabian populations and since has been the focus of many genetic and translational studies in PD [87, 88]. Consequences of the G2019S mutation include gain-of-function increase in LRRK2 protein kinase activity and ubiquitination [89], increased glutamatergic synapse activity and synaptic vesicle release [90], decreased levels of neurotrophic protein progranulin in microglia and fibroblasts [91], a protein linked to neurite growth and neuronal survival [92], lysosomal aggregation [93], and inhibition of autophagic function in dopaminergic neurons [94, 95]. As a whole, G2019S related dysfunction affects synaptic transmission, autophagy, and neurite health in the pathological LRRK2-PD association.

The G2019S-cancer link is disputed by epidemiological studies [96–100] but is starting to be elucidated in the laboratory. In cultured neuroblastoma cells, the G2019S mutant protein initiated the formation of α-synuclein aggregates, propagation into neighboring cells, and subsequent cellular toxicity [101]. G2019S also activates the oncogenic MET/ERK pathway, which was demonstrated to increase basal autophagy and α-synuclein aggregation [102, 103]. These preliminary findings demonstrate potential in finding site-specific targets on LRRK2 as therapeutic options for PD and suspected positively associated cancers, such as breast and prostate.

### Ataxia telangiectasia mutated (*ATM*) and *p53*

*ATM* provides another close genetic link between neurodegeneration and cancer through genome integrity maintenance and cell cycle control. Direct evidence is found in the rare genetic disease ataxia telangiectasia (AT) caused by mutations of *ATM*. AT is characterized by both severe cerebellar degeneration and predisposition for cancer and radiation sensitivity [104, 105]. The gene *ATM* encodes a serine/threonine protein kinase, a member of the phosphatidylinositol-3 kinase (PI3K) superfamily. ATM is recruited initially to the sites of DNA lesions, such as double strand breaks, and phosphorylates several key proteins such as checkpoint kinase CHK2, histone H2AX, the Rad50–Mre11–Nsb1 complex, tumor suppressors p53 and BRCA1, leading to the activation of DNA damage checkpoints, cell cycle arrest, DNA repair, or apoptosis [106–109]. Cells without any functional ATM protein are hypersensitive to radiation and do not respond normally to DNA damage; instead of activating DNA repair, the defective ATM protein allows mutations to accumulate in other genes. *ATM*-heterozygous germline mutations were shown to contribute to breast cancer, non-Hodgkin’s lymphomas and B-cell chronic lymphocytic leukemia, while *ATM*-homozygotes develop lymphoma and leukemia [110, 111]. Because ATM is an essential component of the DNA damage response and apoptosis, its normal expression is crucial for post-mitotic neurons. Camins et al. found that in cells treated with MPP^+^ as an experimental model of PD, cell viability was decreased through activation of ATM and possibly retinoblastoma protein, ultimately leading to initiation of apoptosis [112]. Inhibition of MPP^+^ induced ATM activation displayed neuroprotective effects and increased cell survival [112], although the finding is contradicted by another study showing reduced ATM activity causes neuronal death [113]

ATM can activate repair proteins in response to DNA double-strand breaks, but when the DNA repair machinery is overwhelmed, ATM can also activate p53 to induce cell-cycle arrest and apoptosis. p53 has been recently shown to be involved in aging and neurodegenerative disorders. In PD, p53 levels are increased, possibly through s-nitrosylation of parkin [114], where it mediates microglial activation and subsequent pro-inflammatory phenotypes, leading a p53-driven microglial-evoked neurotoxicity [115–119]. In addition, interaction of p53 with α-synuclein is purported to suppress *Notch1* signaling, leading to retarded neurogenesis in mature neurons [120]. Inhibition of p53 prevents cell death [116, 118, 121, 122].

### Phosphatase and tensin homolog (*PTEN*) and putative kinase 1 (PINK1)

*PTEN* was first identified as a tumor suppressor gene located on chromosome 10q23. PTEN removes the 3′phosphate from phosphatidylinositol 3,4,5-triphosphate (PIP3) and eventually shuts down PI3K-Akt-mTOR pathway leading to growth inhibition and apoptosis [123]. Frequent inactive mutations of *PTEN* were detected in a variety of human cancers including glioblastoma, advanced prostate, and endometrial cancers, and reduced expression is found in many other tumor types such as lung and breast cancer [124–129].

While its role as tumor suppressor is established unambiguously, emerging evidence shows that the biological function of PTEN extends to the CNS where it is widely expressed and localized to the cytoplasm and nucleus [130, 131]. Several studies indicate that PTEN is a crucial regulator of neuronal development, neuronal survival, axonal regeneration, and synaptic plasticity and has been linked to the pathogenesis of neurodegenerative disorders at the molecular level [132–137]. The main target of PTEN, mTOR, plays an essential role in maintaining the integrity of postmitotic neurons by regulating key cellular processes such as protein synthesis, autophagy, mitochondrial metabolism, and biogenesis [138]. The selective inhibitor of mTORC1, rapamycin was shown to be neuroprotective in experimental models of Alzheimer’s disease and PD [139, 140].

PTEN regulates the function of PTEN induced putative kinase 1 (*PINK1*), located on chromosome 1p36, a region frequently deleted in human cancers and mutated in familial forms of PD [141–143]. Similar to another common PD causal gene *PARK2*, PINK1 plays a key role in mitochondrial quality control by identifying damaged mitochondrial and targeting them for degradation, important functions dysregulated in neurodegeneration and cancer [144, 145]. PINK1 is linked with the AKT pathway through PTEN, which is the main driver for tumorigenesis and neuronal survival [145, 146]. Expression of PINK1 mRNA and protein ranges from high to low in different types of cancers, indicating the dual, context dependent pro and anti-tumorigenic role of PINK1 in cancer biology [147]. Flanagan et al. showed that *PINK1* deletion reduced several cancer-related phenotypes like cell proliferation, colony formation and invasiveness through cell cycle control, demonstrating *PINK1* as a potential oncogene [146].

### Melanocortin 1 receptor (*MC1R*)

*MC1R* is the main pigmentation gene that encodes the MC1R protein. A cyclic AMP-stimulating G-protein-coupled receptor, MC1R contributes to regulation of skin physiology though the melanin synthetic pathway. *MC1R* is a genetic determinant of hair color and loss-of-function polymorphisms cause a shift of melanogenesis from the photoprotective, black-brown pigment eumelanin to red-yellow pheomelanin, resulting in a phenotypic spectrum of red hair color, pale skin, and freckles [148]. Epidemiological studies have shown loss-of-function *MC1R* variants are associated with higher risk of developing melanoma [148]. In the laboratory, disruption of MC1R increases oxidative damage and lowers the threshold for melanoma induction even in the absence of UV light [149].

In addition to skin melanocytes, MC1R is expressed in many other cell types including astrocytes [150], Schwann cells [151], and possibly certain neurons of the periaqueductal gray [152] in the brain, suggesting additional functions beyond skin. In two large prospectively followed cohorts, individuals with two copies of the *MC1R* gene loss-of-function variant R151C had a significant three-fold increased risk of developing PD [14]. Newly published findings in an independent cohort substantiate the *MC1R*-PD link via the R160W variant [153]. Another study did not replicate a significant *MC1R*-PD association; however, it also did not replicate the PD-melanoma association and was limited by technical/sequencing difficulty [154]. Together these epidemiological and biochemical findings indicate more general protective role of MC1R in both neurodegeneration and melanoma.

## Other neurodegenerative diseases and cancers

Although the epidemiologic evidence of Alzheimer’s disease (AD)-cancer link is not as intensive as PD-cancer, an inverse association is indicated by several studies. In a prospective cohort study of individuals aged 65 and older, there was 69 % reduced risk of all cancers in AD patients [155]. Romero et al. reported cancer mortality hazard ratio of 0.5 in people with AD versus no dementia [156]. In specific cancers, there is significant 40 % decrease in risk of epithelial and lung cancers and 57 % decrease in risk of colorectal cancer in people with AD [157]. In a gender and age matched case control study, cancer had significant inverse association with AD only in women and endocrine-related tumors with odds ratios of 0.5 for both, although their study suggested overall inverse association with all types of cancers [158].

The association between AD and cancer is also bidirectional. Driver and et al. found a hazard ratio of 0.67 comparing 1278 study participants with and without cancer [159]. In population-based longitudinal study of aging including 1100 people aged 70 or older, non-melanoma skin cancer history is associated with about 79 % less AD risk [160]. Likewise, a study from the Alzheimer’s Disease Neuroimaging Initiative provides evidence that a history of non-melanoma skin cancer, but not breast or prostate cancer, has inverse association with AD diagnosis [161], contrary to the positive association observed in PD patients. Intriguingly, although cancer survivors have lower risk of AD, reduced cognitive function and cerebral gray matter density were also found in cancer patients pre and post-treatment, which complicates the apparent reverse link [161].

Although the biological evidence is scarce, the infamous *PTEN* gene and protein of PD has a role in AD progression. A significant loss and alteration of PTEN was found in AD neurons [162] and its downstream targets may explain its pathological significance. Glycogen synthase kinase regulates tau phosphorylation [163] while AKT mediates neuronal survival against β-amyloid neurotoxicity in experimental models of AD [164, 165].

We discussed the most researched biological mechanisms associated with PD genes, but they are not an exhaustive review. A brief summary of the genes in neurodegenerative diseases and some of their pathological effects are listed in Table 3. Table 4 lists genes in cancer that have been implicated PD.

**Table 3:** List of representative neurodegeneration-associated genes and cancer

Gene/Protein	Biological functions	Changes in neurodegeneration	Implicated cancers
*SNCA*/Alpha-synuclein	synaptic vesicle and dopamine release [174, 175]; excitatory transmission [176]; endoplasmic reticulum-Golgi transport [177]	major constituent of Lewy bodies; impaired neurite growth and long-term potentiation [178]; increased synaptic transmission and endoplasmic reticulum stress [177]; increased gliosis [179]; increased mitophagy [180]	adenocarcinoma, lung [181]; colorectal [182]; brain [183]; melanoma [184]; prostate [185]; non-Hodgkin lymphomas [51]
*PARK8*/LRRK2	synaptic vesicle release [186]; autophagy [187]; neurite growth and differentiation [188]; cell death signaling [189]; mitochondrial regulation [190, 191]; cytoskeletal structure maintenance [192]	increased tau phosphorylation [193]; mitochondrial and autophagic dysfunction [194]; decreased neurite outgrowth and abnormal neurogenesis [195]	breast, prostate [96, 97]; renal, thyroid [102]
*PARK2*/Parkin	synaptic transmission and dopamine release [196]; ubiquitination and protein degradation [197]; mitochondrial maintenance [198]; tumor suppressor [199]	mitophagy, mitochondrial transport and morphology defects [200]; dysfunctional UPS [60]; buildup of cyclin E and β-catenin, upregulation of Wnt and EGFR-AKT pathways [61, 73]	cervical, lung, colorectal, gastric, melanoma, endometrioid [70]; glioma [73]
*PARK6*/PINK1	serine/threonine kinase in mitochondria; mitochondrial fusion/fission regulation [201]; mitochondrial damage sensor, mitophagy and autophagic control [198]; cell cycle regulation [146]; synaptic plasticity and dopamine release [202]	increased tau phosphorylation [203]; mitochondrial dysfunction, fragmentation [204]; increased mitophagy [205]; impaired synaptic plasticity [202]	breast [206]; glioma, ovarian [207]
*PARK7*/DJ-1	oxidative stress protection [208]; redox-sensitive protein chaperone [209]; transcriptional regulation, mitochondrial regulation [210–212]	increased oxidative stress sensitivity [213]; reduced complex I activity in mitochondria [214]; increased tau phosphorylation [215]	breast [216]; lung [217]; pancreatic [218]; gastric [219]; prostate [220]
*MAPT*/Tau	microtubule-associated protein, tubulin polymerization, scaffolding protein [221, 222]; growth factor signaling [222]; synaptic regulation [223]	hyperphosphorylated tau, major component of neurofibrillary tangles; synapse degeneration [223]	prostate [224]; breast [225]; epithelial ovarian [226]
*APP*/APP	synapse formation and maintenance [227]; trophic activity, neurite growth, axon pruning [228, 229]	mutations lead to Aβ peptide and amyloid plaques [229]	myeloid leukemia [230]; testicular [231]

**Table 4:** List of representative genes in cancer implicated in neurodegenerative diseases

Gene/Protein	Biological functions	Association with Cancers	Roles in neurodegeneration
*TP53*/p53	transcriptional factor, regulates DNA repair, cell cycle control, apoptosis [115]	tumor suppressor [232]	acts as protective factor by interacting with PD or AD related proteins via cell cycle and apoptosis signaling [122]
*ATM*/ATM	serine/threonine protein kinase; regulates DNA repair, cell cycle control, apoptosis [108, 233]	tumor suppressor; mutations increase the risk of breast, stomach, bladder, pancreas, lung, lymphoid and ovarian cancers [110, 234, 235]	mutations lead to AT, inactivation causes cerebellar degeneration [104]
*PTEN*/PTEN	protein tyrosine phosphatase [123]; apoptosis regulation, migration, and adhesion, angiogenesis [129]	tumor suppressor [129]; mutations cause PTEN hamartoma tumor syndrome and increase the risk of breast cancer, prostate cancer and endometrial cancer [124, 127, 132]	promotes axon regeneration [136]; regulates PINK1 and mTOR pathway [136]
*MC1R/*MC1R	main pigmentation gene [148]	loss-of-function variants associated with melanoma [148, 236]	variants R151C and R160W increase PD risk [14, 153]

## Concluding remarks

Although overlap between PD and cancer is becoming evident, there are several concerns stemming from some of the contradictory results on both the epidemiological and laboratory fronts. One, there are undoubtedly differences in genetic mutations across ethnicities and races, as seen in the discrepancies in risk and prevalence of cancers between Eastern and Western populations, and genetic or pharmacological models with clinical applications should note the groups of most likely pertinence. Second, some studies have noted that cancers that were associated with PD approached significance depending on the range of time between the two diagnoses, which was not considered in the majority of studies, highlighting either complex temporal associations or ascertainment and survival bias that should be carefully considered. To eliminate spurious associations, stringent measures to ascertain the time of PD and cancer onset should be employed in study design and selection of available data. Third, more rigorous and cancer specific epidemiological studies are needed to support recent biological evidence linking PD and cancer, as many studies have included highly heterogeneous cancers on all-cancer risk in PD. For example, mutated *PARK2* is expressed in glioblastoma, colon, and lung cancer, but the latter two cancers are associated with decreased risk of PD, contrary to increased risk in glioblastoma [3, 13, 69]. Whether or not the disparities arise from divergent pathways in different tissues is still relatively unknown, although pathological clues from neurodegenerative diseases applied to linked cancers have gained much interest due to strides in epidemiological research.

The associations between neurodegeneration and cancer presented here are likely a fraction of the plethora of shared mechanisms of these two distinct disorders. Further investigations of these links and shared genetic determinants implicated in these pathways may offer valuable perspectives and new therapeutic options for the two groups of traditionally disparate yet pathologically convergent diseases.
